# A Degradable and Self-Healable Vitrimer Based on Non-isocyanate Polyurethane

**DOI:** 10.3389/fchem.2020.585569

**Published:** 2020-10-16

**Authors:** Haitao Wu, Biqiang Jin, Hao Wang, Wenqiang Wu, Zhenxing Cao, Jinrong Wu, Guangsu Huang

**Affiliations:** State Key Laboratory of Polymer Materials Engineering, College of Polymer Science and Engineering, Sichuan University, Chengdu, China

**Keywords:** vitrimer, non-isocyanate polyurethane, acid-degradation, strain-rate response, self-healing

## Abstract

Developing degradable and self-healable elastomers composed of reusable resources is of great value but is rarely reported because of the undegradable molecular chains. Herein, we report a class of degradable and self-healable vitrimers based on non-isocyanate polyurethane elastomer. Such vitrimers are fabricated by copolymerizing *bis*(6-membered cyclic carbonate) and amino-terminated liquid nitrile rubber. The networks topologies can rearrange by transcarbonation exchange reactions between hydroxyl and carbonate groups at elevated temperatures; as such, vitrimers after reprocessing can recover 82.9–95.6% of initial tensile strength and 59–131% of initial storage modulus. Interestingly, the networks can be hydrolyzed and decarbonated in the strong acid solution to recover 75% of the pure di(trimethylolpropane) monomer. Additionally, the elastomer exhibits excellent self-healing efficiency (~88%) and fracture strain (~1,200%) by tuning the monomer feeding ratio. Therefore, this work provides a novel strategy to fabricate the sustainable elastomers with minimum environmental impact.

## Introduction

Elastomers are a class of the most important polymer materials that have diverse applications in automobile, biomedical, and aerospace industries, as well as our daily life. Traditional covalently cross-linked elastomers display excellent mechanical properties and thermal and chemical stability (Tee et al., 2012; Tadakaluru et al., 2014; Wang and Loh, 2017). However, the irreversibility of chemical network prevents the recycling, reprocessing, and degradability of elastomer products, which puts a huge burden on ecology and environment.

One effective way to address this issue is to introduce dynamic covalent bonds into elastomer networks (Scott et al., 2005; Jin et al., 2013; Kloxin and Bowman, 2013; Garcia and Smulders, 2016; Chen et al., 2019; Jiang et al., 2020). The resulting dynamic networks can rearrange their topologies via exchange reactions under external stimuli, which imparts the elastomers with reprocessability and damage healability. Among various dynamic chemistries, vitrimers, a concept pioneered by Röttger et al. is particularly attractive because of associative topological rearrangement of the dynamic covalent networks (Montarnal et al., 2011; Röttger et al., 2017). As such, they hold huge potential in a variety of applications, such as recyclable thermoset polymers and self-healable polymers (Yang et al., 2014, 2016; Ube et al., 2016; Shi et al., 2017; Zhang and Xu, 2017). The fascinating performance of vitrimer was first demonstrated in a carboxylic acid cured epoxy network, which undergoes transesterification under catalytic Zn(OAc)_2_ (Montarnal et al., 2011). Currently, a variety of strategies have been adopted to design vitrimer or vitrimer-like materials, including carboxylate transesterification (Cromwell et al., 2015; Chen et al., 2018; Liu et al., 2018; Wang et al., 2019), transalkylation of triazolium salts (Obadia et al., 2015), transcarbamoylation (Fortman et al., 2015), olefin metathesis (Lu et al., 2012), boronic ester exchange (Röttger et al., 2017), disulfide exchange (Jian et al., 2018; Zhou et al., 2018), siloxane silanol exchange (Zheng and McCarthy, 2012), transamination (Denissen et al., 2017), etc. These strategies impart the vitrimer materials with reprocessable, recyclable, and repairable properties via bond exchange reactions at elevated temperatures; however, the products are usually non-degradable and insoluble, which causes serious ecological and environmental pollution at the end of the service life. Therefore, developing a degradable and self-healable vitrimer material is of great public value.

**Figure 1:**
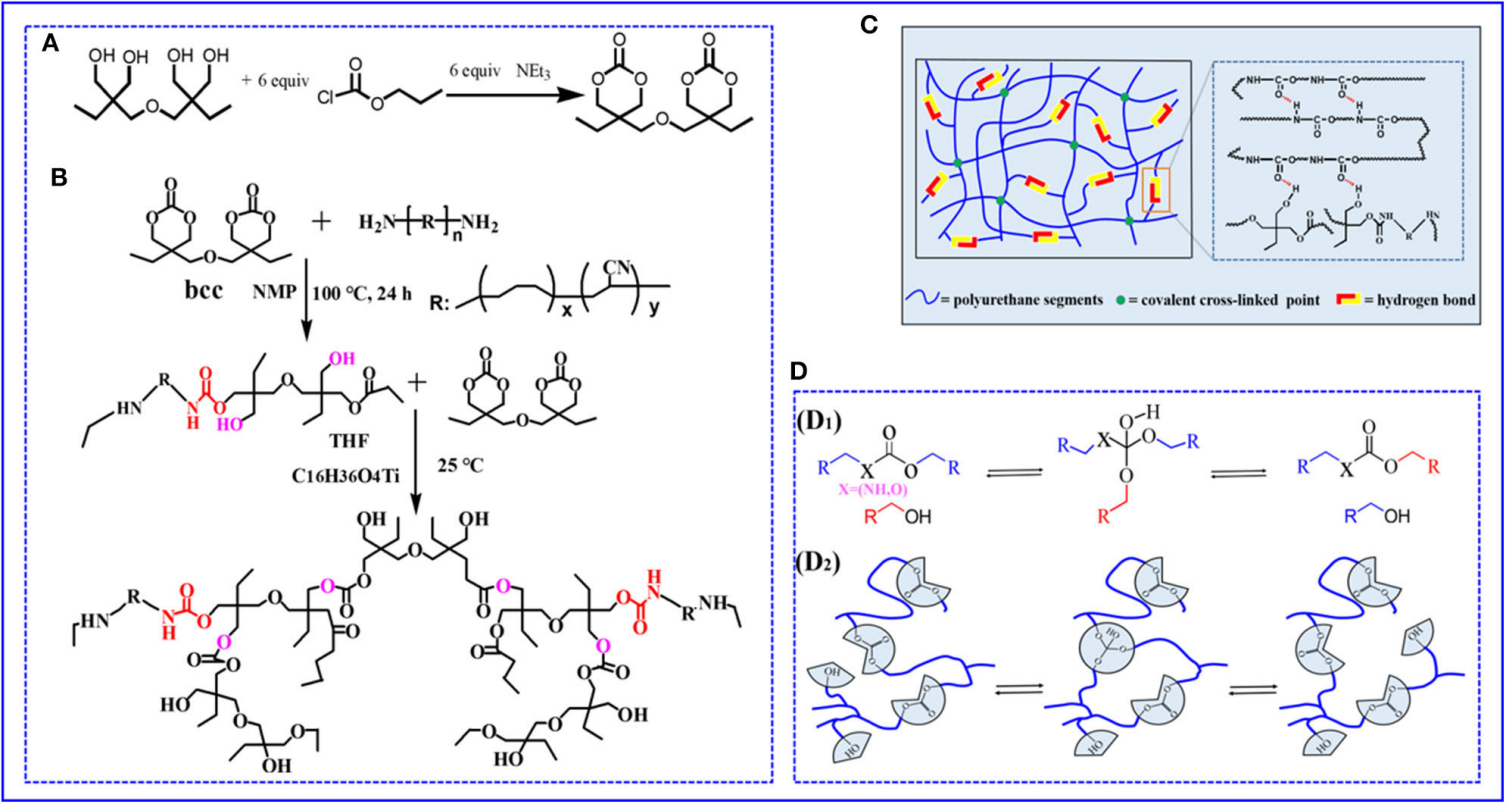
**(A,B)** The chemical synthesis route and molecular design of non-isocyanate PU_E_ **(C)** Cartoon representation of the proposed cross-linked network structure for PU_E_ **(D)** Reprocessing mechanism of non-isocyanate PU_E_ (D_1_). Transcarbonation exchange reaction whereby a hydroxyl nucleophile reacts with a carbonate, forming an associative intermediate, and releasing the exchanged carbonate and hydroxyl group. (D_2_) Topology of a cross-linked elastomer network containing carbonates and hydroxyls can be adjusted via transcarbonation exchange reactions.

In this article, we develop a novel and efficient path to prepare a degradable and self-healable vitrimer based on non-isocyanate polyurethane elastomer (PU_E_) by introducing carbonates that can undergo transcarbonation exchange reactions in Figure 1. The prepared elastomer can rearrange the network topology via transcarbonation exchange reactions, thus endowing the elastomer with vitrimer behaviors. In addition to being reprocessable and repairable, the networks can be hydrolyzed and decarbonated in hydrochloric acid solution to recover the di(trimethylolpropane) monomer and Pre-PU_E_.

## Experimental

### Materials

Amino-terminated liquid nitrile rubber (ATBN, Mn = 402 g mol^−1^) was purchased from Shandong Jining benoke Biotechnology Co., Ltd. Chloroformic acid–propylester (98%), n-methyl pyrrolidone (NMP), anhydrous tetrahydrofuran (THF), and di-trimethylolpropane (98%) were provided by Shanghai TITAN Technology Co., Ltd. Ethanol (AR grade), butyl titanate (C_16_H_36_O_4_Ti), and triethylamine (Et_3_N, AR grade) were supplied by Kelong Reagent Corp. (Chengdu, China). All of the raw materials were dried before use.

### Synthesis of *bis*(6-Membered Cyclic Carbonate) (Bcc)

Bcc was successfully synthesized from di(trimethylolpropane) and chloroformic acid–propylester reaction procedure in Figure 1A. The details of the preparation were as follows: di-trimethylolpropane (10.0 g, 40 mmol) was dissolved in THF (250 mL) and transferred to three-neck round bottom flask with magnetic stirring 1 h under N_2_ at 25°C, and then chloroformic acid–propylester (26 mL) was step-by-step drop added into a three-neck flask during stirring. Subsequently, catalyst Et_3_N was added dropwise when the reaction temperature cooled to 0°C. Then, the reaction mixture was magnetic stirring for 5 h to form a white precipitate and removed by vacuum filtration, and solvent was removed via under rotary evaporation to yield a white solid. Finally, the white solid was recrystallized in THF (10 mL) to attain the pure product Bcc.

### Preparation of Polymer PU_E_

Attributing to the long molecular chain structure of ATBN, the end amino groups in long-chain molecule were surrounded by ATBN molecular chains, leading to a limited reactivity between amino and *bis*(6-membered cyclic carbonate). To improve the reactivity of the reaction, the low-molecular-weight ATBN was first chosen. Bcc (0.5 g, 1.65 mmol) and ATBN (29 g, 60 mmol) were added into three-neck round bottom under nitrogen atmosphere. Then, the reaction solvent NMP (60 mL) was added to the mixture, after nucleophilic addition reaction at 100°C with magnetic stirring for 24 h and formed prepolymer. Bcc can serve as cross-linker to realize cross-linking existed catalyst. For details, the cross-linked PU_E_ was synthesized by a one-step copolymerization existed catalyst C_16_H_36_O_4_Ti and THF at 25°C. Finally, the polymer PU_E_ was successfully prepared (Figure 1B). Because of existed substantial hydrogen bonds formed by hydroxyl and carbonate groups, dual cross-linked networks were thus formed during polymer preparation process in Figure 1C. Because the content of cross-linker Bcc and physical cross-links directly influences the polymer crosslinking density and thus has impact on the mechanical properties, a series of samples were synthesized by tuning the feed ratio. The feed ratios of PU_*E*_—*X* (*X* = 1, 2, 3, 4, 5) are summarized in Supplementary Table 1.

### Preparation of Polymer PU_E_ Film

The product PU_E_ solution was first put into a Teflon mold (30 × 50 × 10 mm) with a glass plate and was subsequently placed in an oven for 12 h at 40°C. Second, the product solution was heated from 40 to 50°C for 12 h. Additionally, the resulting polymer film was dried under vacuum at 50°C for 24 h. Finally, the film was taken out and cut into rectangles of 2 × 25 mm for further testing.

### Characterization Methods

Prior to this, polymer PU_E_ samples were vacuum-dried at 50°C for 12 h.

#### Fourier Transform–Infrared Spectrometry (FT-IR)

FT-IR spectral analysis was recorded on a Fourier transform infrared spectrometer (Nicolet iS50) in a range of wavenumbers from 4,000 to 500 cm^−1^.

#### Nuclear Magnetic Resonance Spectroscopy (NMR)

NMR spectra were recorded on a Bruker AV400 spectrometer (400 MHz, Germany) with dimethyl sulfoxide-*d*_6_ and deuterated chloroform as the solvents and tetramethyl silane as the internal reference.

#### Dynamic Mechanical Analysis (DMA)

The dynamic mechanical analysis was studied using DMA Q800 (TA Instruments, USA), at a frequency of 1 Hz and then a heating rate of 3°C min^−1^ from −100 to 100°C.

#### Differential Scanning Calorimetry (DSC)

DSC was performed with a DSC-Q200 (TA Instrument, USA) over the temperature range from −60 to 60°C at a heating rate of 5°C min^−1^ under N_2_ and empty aluminum as the reference.

#### Scanning Electron Microscope (SEM)

A scanning electron microscope (Nova NanoSEM450) was used to trace the healing process of scratch immediately after making scratch by a blade.

#### Static Tensile Tests

The mechanical properties of the samples were investigated on an Instron Universal Testing Machine (Model 5967, Instron Crop) at a stretching rate of 0.083 s^−1^ at room temperature. The thickness and width of the specimens were 0.70 and 4 mm, respectively. The length of the sample between the two pneumatic grips of the testing machine was 20 mm. Three dumbbell-shaped specimens were tested in each sample.

#### Acid-Degradation Studies

To conduct swelling test, 0.5-g sample of polymer film was submerged in THF (15 mL) and then stirred for 24 h at room temperature until no obvious changes appear. It was shown that the cross-linked network only was swollen. For degradation studies, the same 0.5-g PU_E_ sample films were immersed in sealed vials containing 10 mL of HCl (1 M), KOH (1 M), and H_2_O, respectively. The vial was heated in a drying oven to 100°C for 24 h. The sample was cleaned containing HCl solution with n-butanol (20 mL). The solvent was removed by rotary evaporation, and finally, the di(trimethylolpropane) monomer was obtained.

#### Reprocessing Recovery

To reprocess the PU_E_ materials, the sample films were ground to small pieces first, and second placed in a hot press at 130°C under the pressure of 5 to 10 MPa. These pieces were thermally equilibrated for 30 min and removed from the press to check for homogeneity of the film and then once again placed in a hot press at 130°C for 2 h under same pressure. Finally, uniaxial tensile tests and dynamic mechanical thermal analysis were carried out to determine the recovery efficiency of mechanical properties.

#### Self-Healing Tests

The elastomer sample was cut into two pieces at the center using a razor blade; the two pieces rejoin together to form nearly complete elastomer when contacting with each other at 100°C. Uniaxial tensile test was performed to determine the mechanical properties and healing efficiencies.

### Polymer Characterization

The chemical structure of Pre-PU_E_ and PU_E_ samples was characterized using FT-IR spectra, as shown in Supplementary Figure 3. It can be found that the prepolymer sample exhibits several characteristics peaks: the absorption peak at 3,840 cm^−1^ indicates the presence of –OH; the peaks at 2,980 cm^−1^ can be attributed to the N-H bending vibration of the carbamate groups (–NHCOO) (Fan et al., 2019), and the peaks at 1,460 and 1,741 cm^−1^ can be assigned to the C–O stretching vibration and C = O stretching vibration of linear carbonate, respectively (Snyder et al., 2018). These results illustrated that the presence of the carbamate and carbonate groups. It can be also seen that Pre-PU_E_ and PU_E_ samples basically showed similar FT-IR spectra. Swelling tests were performed; this Pre-PU_E_ and PU_E_ samples of 1.5 g were immersed in 10 mL THF for 36 h at room temperature in Supplementary Figure 3, respectively. The Pre-PU_E_ was dissolved, whereas PU_E_ is only swollen, which indicates that the PU_E_ is cross-linked under catalyst C_16_H_36_O_4_Ti. These results suggest that the polymer PU_E_ was successfully prepared. Additionally, the temperature-dependent FT-IR spectra of PU_E_ were collected, as shown in Supplementary Figure 4. Upon heating from 10 to 150°C, the peak intensity at 1,741 cm^−1^ weakened, whereas the peak at 1,620 cm^−1^ (free urea C = O) intensified, which indicated that increasing the temperature can cause gradually dissociation of hydrogen bonds, which was consistent with the previously reported literature (Liu et al., 2019).

## Results and Discussion

### Thermal Properties and Dynamic Nature of the Networks

**Figure 2:**
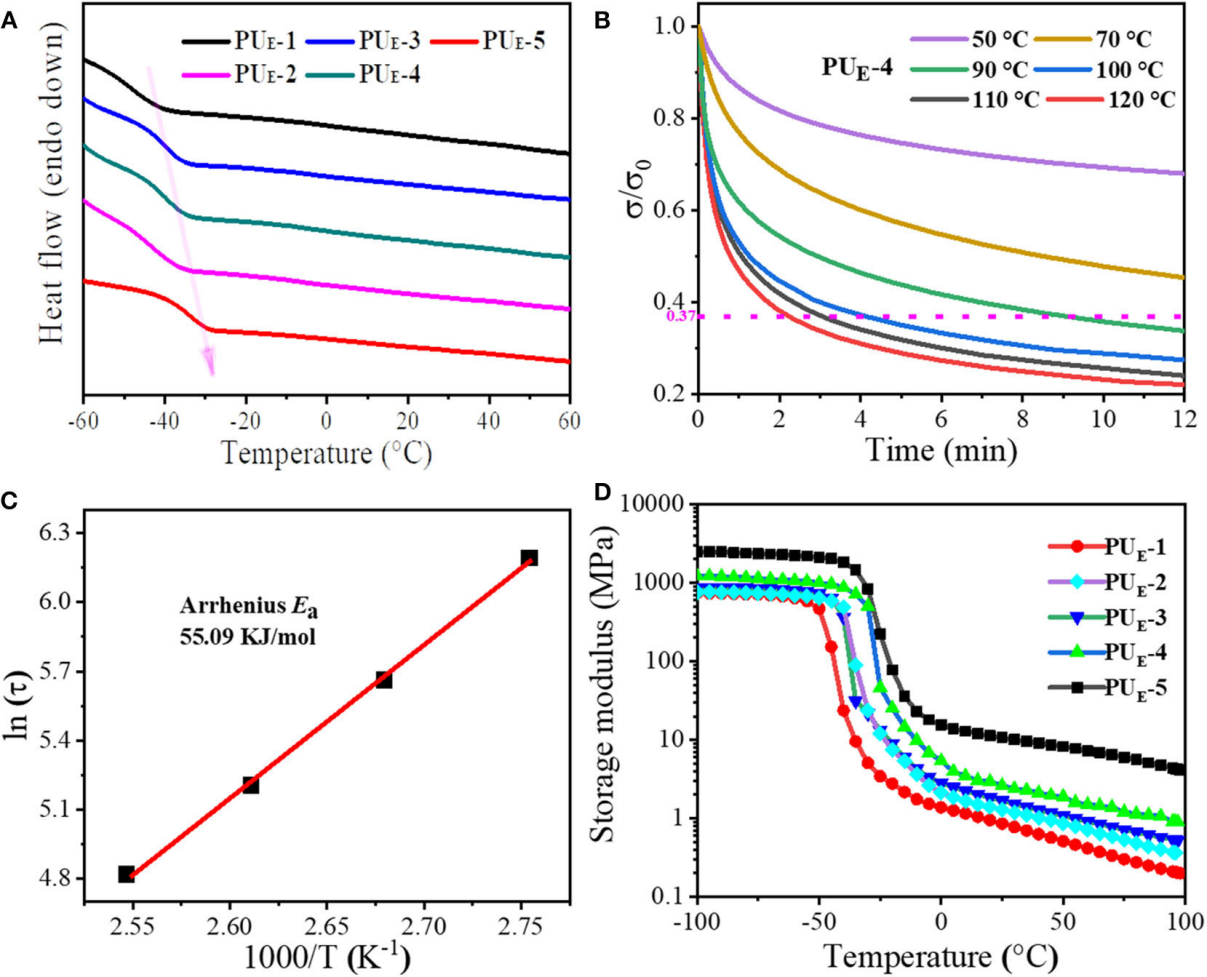
Thermal properties and dynamic nature of the networks. **(A)** DSC curves of PU_E_ samples under N_2_ in the temperature range of −60 to 60°C at a heating scan rate of 5°C min–^1^. **(B)** Stress relaxation curves of PU_E_-4 sample. The sample is stretched to a strain of 50% under different temperatures from 50 to 120°C and then maintained for 12 min. **(C)** Fitting of the relaxation times to the Arrhenius equation. **(D)** Dependence of the PU_E_ samples storage modulus *(E*′*)* on temperature.

The thermal properties of polymer materials play a dominant role in their topological rearrangement of networks. Therefore, differential scanning calorimetry (DSC) is performed. Figure 2A displays the DSC curves of PU_E_ samples. The relevant thermal parameters and their corresponding values are summarized in Supplementary Table 2. It is evident that the glass transition temperature (*T*_*g*_) of PU_E_ samples increases with the increasing cross-linker Bcc amount. This suggests that the enhancement of the degree of cross-linking greatly restricts the mobility of chain segments, as shown in Supplementary Figure 5A. As is expected, there is no endothermic peak above *T*_*g*_, which indicates that the material is amorphous. In addition, it can be found from Supplementary Figure 5A that the *T*_*g*_ values are lower than room temperature, indicating the material is an elastomer rather than plastic at room temperature. Meanwhile, thermogravimetric analysis measurements are conducted, and the values of thermal degradation temperatures are summarized in Supplementary Table 2. The elastomer network first lose mass around 214°C (5% mass loss), which is well above the temperature required for transcarbonation exchange reaction in Supplementary Figure 5B.

To better determine the network topology rearrangement via transcarbonation exchange reactions between free hydroxyl and carbonate groups at elevated temperatures and the rate of exchange reactions, the stress–relaxation measurements are performed by stretching the samples to a strain of 50% from 50 to 120°C. The strain is then maintained for 12 min, and the stress is recorded as a function of time in Figure 2B. The network characteristic relaxation time (τ) is defined as the time required for the elastomer to reach *1/e* (37%) of the initial stress and is used as a measure of the rate of transcarbonation exchange reactions under the testing conditions (Snyder et al., 2018). As illustrated by Figure 2B, it can be seen that PU_E_-4 has a stress relaxation time of 547 s at 90°C. As the temperature increases to 120°C, the relaxation time decreases to 132 s. The result demonstrates that the exchange rate of elastomer network rearrangement largely depends on the temperature. As shown in Figures 2B,C, the temperature dependence of the relaxation time of PU_E_-4 follows Arrhenius' law (Wu et al., 2020). According to Arrhenius' law:

(1) $$\begin{array}{l} \text{ln}\tau =\ln\tau_{0}+\frac{E_{a}}{RT} \end{array}$$

The activation energy of the rearrangement network is determined to be 55.09 kJ mol^−1^. As the temperature rises, the adaptivity of the PU_E_-4 network is activated as a result of exchange reaction. Simultaneously, the samples with different cross-linker amount are fixed at 100°C and then maintained for 12 min; it can be seen that the residual stress of these samples lower than *1/e* of the initial stress undergoes stress relaxation within 4 min, illustrating that the network topologies of all these samples can be rearranged at elevated temperature in Supplementary Figure 6. Moreover, the storage modulus (*E*′) and the loss factor (tanδ) of the elastomers as a function of temperature are recorded by DMA, as shown in Figure 2D and Supplementary Figure 5D. The samples exhibit a marked decrease of *E*′ when the temperature exceeds *T*_*g*_ in Figure 2D. Interestingly, the rubber modulus decreases with temperature, indicating the dissociation of hydrogen bonds prior to dynamic network change. Not surprisingly, the sample with the higher cross-linker Bcc content possesses a higher *E*′-value. According to the *E*′ value at *T*_*g*_**+** 30°C (Tang et al., 2020), the cross-linking density can be calculated in Supplementary Equation 1 and shown in Supplementary Table 2.

### Mechanical Properties

**Figure 3:**
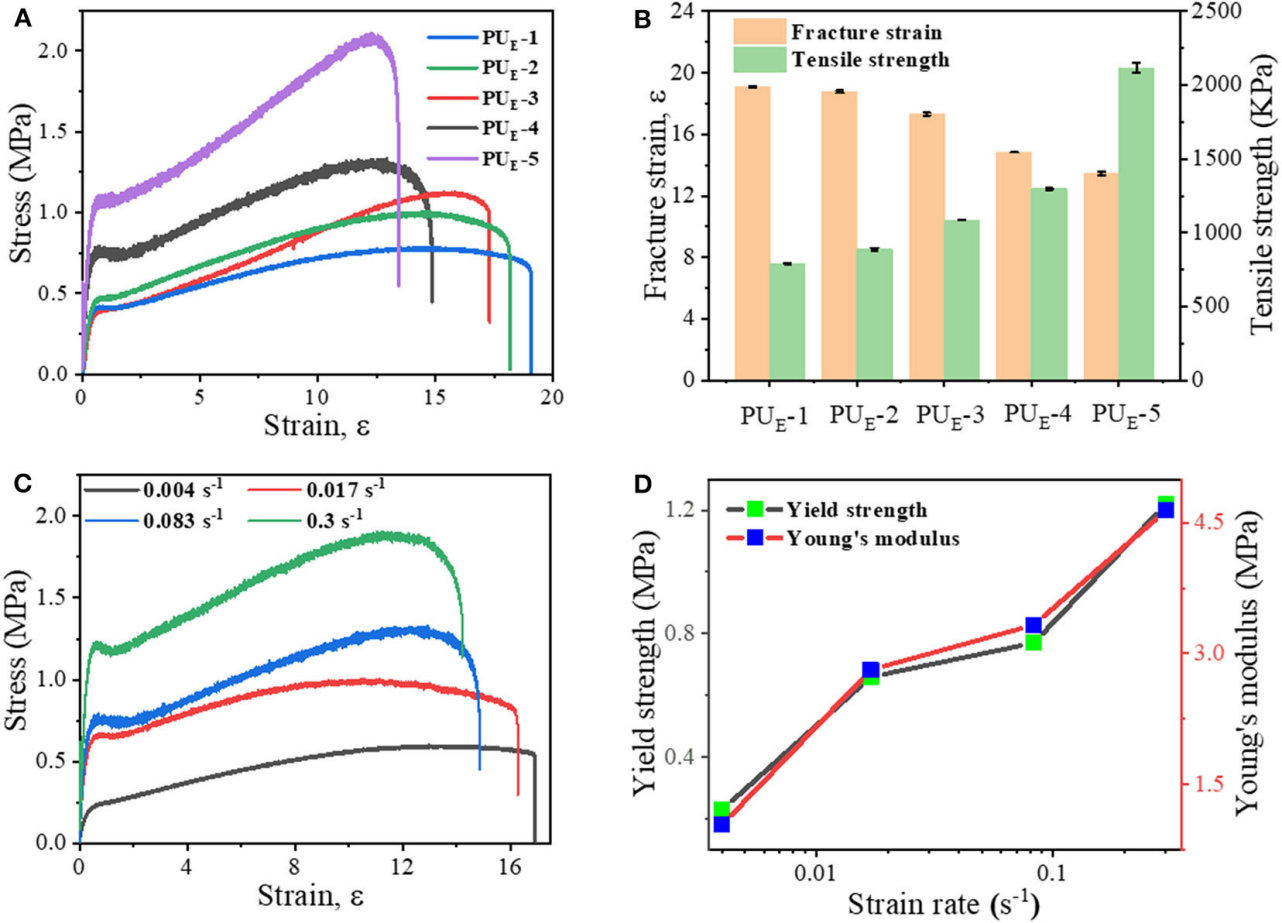
Static tensile properties of the elastomers. **(A)** Tensile tests of as-prepared samples with various feeding ratios: the strain rate is 0.083 s^−1^. **(B)** Summary of mechanical properties of the elastomers. **(C)** Stress—strain curves of the PU_E_-4 sample at various strain rates. **(D)** Dependence of the yield strength and Young's modulus on the various strain rates.

**Figure 4:**
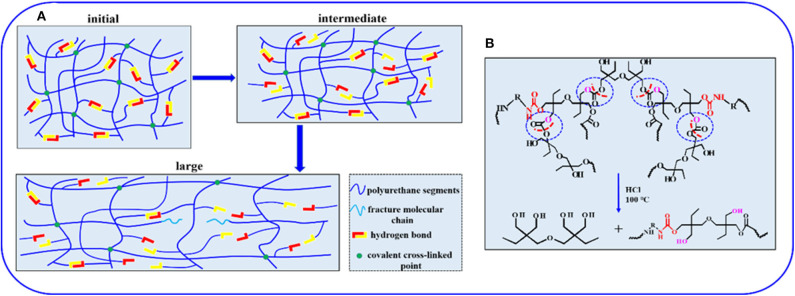
**(A)** Illustration of the sequential breakage of networks in the elastomer during the stretching process. **(B)** The acid-degradation mechanism of non-isocyanate PU_E_.

To investigate the mechanical properties of the elastomer, static tensile measurements are performed at a strain rate of 0.083 s^−1^. Figure 3A displays the typical stress–strain curves for these samples, and mechanical properties are summarized in Figure 3B and Supplementary Table 4. PU_E_-1 exhibits a tensile strength about 0.79 MPa and a strain at break of 1,696%, as the cross-linker Bcc content increases to 0.519 g (1.720 mmol), the tensile strength increases up to 2.09 MPa. The improved mechanical properties can be attributed to the synergistic effect of covalent cross-links and physical cross-links formed by substantial hydrogen bonds. The covalent bonds control the network elasticity and maintain the sample integrity at large deformation, whereas the recurrent dissociation/reassociation of the hydrogen bonds controls rigidity and toughness. Thus, when stretched, the hydrogen bonds fracture first to dissipate energy effectively, while the covalent bonds maintain a good strength of elastomer, as illustrated by Figure 4A. Interestingly, the stress–strain curves of these samples are relatively non-linear, and there is yielding point, which represents a non-classical rubberlike behavior.

To further explore the non-linear relationship between deforming and mechanical properties, we selected PU_E_-4 as an example to perform the tensile tests at various straining rates, from 0.004 to 0.3 s^−1^. Figure 3C manifests a unique strain-rate–responsive yield phenomenon. The yield stress strikingly with increasing strain rate, and meanwhile the Young's modulus obtained from the initial linear region of the stress–strain curves increases from 1.04 to 4.64 MPa in Figure 3D. Such a phenomenon demonstrates that the elastomers possess a self-stiffening ability with increasing strain rate (Wu et al., 2019). To explain the underlying mechanism governing this increasing yield phenomenon with strain rate, we need to consider the inherent (stress-free) dissociation rate constant (*k*_*p*_) of the hydrogen bonds (Hu et al., 2017). At room temperature, *k*_*p*_ of the hydrogen bonds is much smaller than the strain rate. As a result, higher strain rates (>0.004 s^−1^) tend to enhance the strain localization and thus promote yielding, which induces a slight drop in stress.

Additionally, the stretchability is highly dependent on the straining rate in Supplementary Figure 7, which is consistent with most elastomers (Li et al., 2016; Son et al., 2018; Lai et al., 2019). Surprisingly, unlike other super-stretchable elastomers with large residual strain after deformation, the non-isocyanate PU_E_ displays an excellent elastic behavior and can nearly completely restore its original shape even after they are subjected to large deformations. For an example, PU_E_-4 can be stretched to a strain of 1,700%. After releasing the stress, the instantaneous residual strain is 70%, and it gradually decreases as a function of time, as shown in Supplementary Figures 8A,B. Prolonging the waiting time, the sample almost fully recovers its original shape without evident residue strain, which suggests both the robustness of the covalent network and reversibility of the physical network formed by hydrogen bonds. The performance comparison between this material and the existing literatures PU elastomer has been put into the (Supplementary Figure 9). (Yuan et al., 2014; Chen et al., 2016; Feula et al., 2016; Yang et al., 2017, 2020; Jin et al., 2020).

### Acid-Degradation and Reprocessing

**Figure 5:**
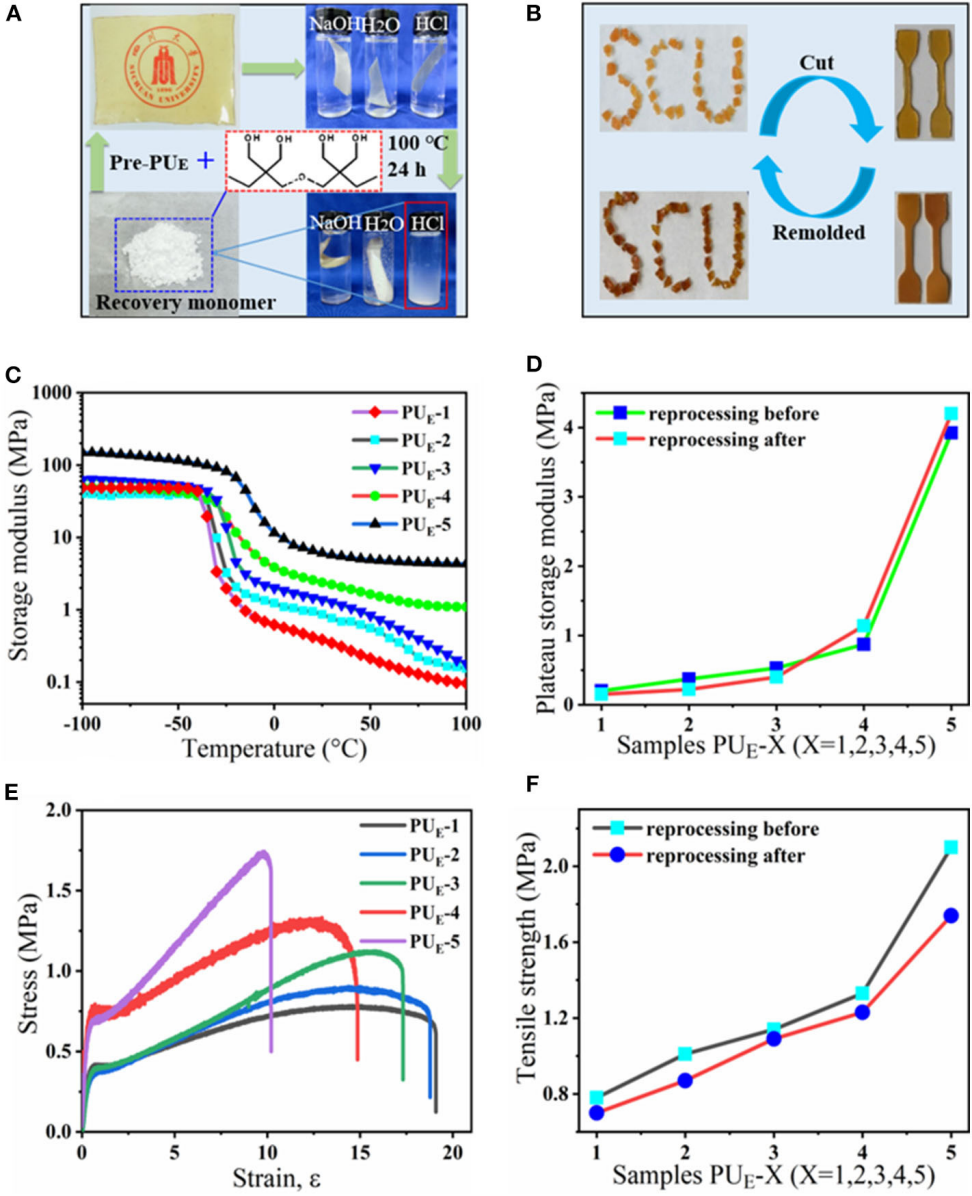
The acid-degradation and reprocessing of the PU_E_ samples. **(A)** A sample film is hydrolyzed and decarbonated in hydrochloric acid, and the monomer can be recovered. **(B)** Hot reprocessing of the damaged sample films. **(C)** DMA curves of the samples after reprocessing. **(D)** The variation curves of the rubbery storage modulus at 50°C before and after reprocessing. **(E)** Stress-strain curves of the samples after reprocessing. **(F)** The variation curves of the tensile strength before and after reprocessing.

Compared with traditional isocyanate-based PU_E_s, one degradation process allows the PU_E_ to recover the di(trimethylolpropane) monomer under acid hydrolysis condition at the end of service life. To illustrate the degradation process, the sample films of 0.5 g are immersed in sealed vials containing 10 mL of HCl (1 M), NaOH (1 M) and H_2_O, respectively. The vials are heated in an oven to 100°C for 24 h. The sample films show no obvious changes in aqueous solution (H_2_O) and slight degradation in basic solution (NaOH), while in strong acid solution (HCl), the sample can be fully dissolved into a homogenous solution after heating, as shown in Figure 5A. The acid-degradation mechanism is shown in Figure 4B: the carbonate bonds are broken in the strong acid solution. Extracting the acid-degraded solution containing HCl with *n*-butanol (20 mL) leads to 75% recovery of the pure di(trimethylolpropane) monomer. The product structure is not destroyed, as verified by NMR characterization in Supplementary Figure 10. In addition to being acid-degradable, the dynamic cross-linked network can reform once again at elevated temperatures owing to transcarbonation exchange reactions via free hydroxyl and carbonate groups. As such, the samples can be reprocessed. For example, the damaged samples are cut into small pieces and then hot pressed at 130°C under a pressure of 5 to 10 MPa. The sample can be fully restored after hot pressing in Figure 5B, indicating the excellent recyclability of the dynamically cross-linked elastomer.

The reprocessing mechanism relies on transcarbonation exchange reaction and is illustrated in Figure 1D: a hydroxyl nucleophile reacts with a carbonate at elevated temperature, forming an associative intermediate, and then the exchanged carbonate and hydroxyl groups are released, denoting completion of the network rearrangement process. Dynamic mechanical analysis and uniaxial tensile tests are carried out to determine the recovery efficiencies of the tensile strength and plateau storage modulus after reprocessing. The storage modulus of the samples after reprocessing as a function of temperature is recorded by DMA in Figure 5C. Figure 5D exhibits the plateau storage modulus at 50°C before and after reprocessing. The performance of the reprocessed material, which was better than its original form. Similar results have been found in previous literature (Snyder et al., 2018), and the mechanism is still unclear. Exploring the questions will require model networks of more controlled network structure and molar ratio between reversible and covalent crosslinks. The typical stress–strain curves of these samples after hot pressing are illustrated by Figure 5E. The stress–strain curves of remolded samples also show the unique yield phenomenon. The tensile strength of the samples, however, decreases as reprocessing process in Figure 5F, suggesting that the reprocessing procedures would cause slightly decreases in tensile strength. Despite the slight decrease of mechanical properties, no changes in the chemical functionality are observed by FT-IR spectra in Supplementary Figure 11. Meanwhile, to quantify the reprocessing tensile strength recovery efficiency, we define it as ratio the tensile strength of before to after reprocessing in Supplementary Equation 2. The recovery efficiency of tensile strength is 82.9–95.6% after reprocessing in Supplementary Figure 12. Recovery of elastomers and avoidance of environmental pollution are the main research directions of soft materials. Although the degradation cost mentioned in this article is not the lowest, relatively speaking, it provides at least a feasible and easy to implement recycling strategy. How to reduce the cost and obtain the highest recovery product is the work we need to continue to carry out.

### Self-Healing Properties

**Figure 6:**
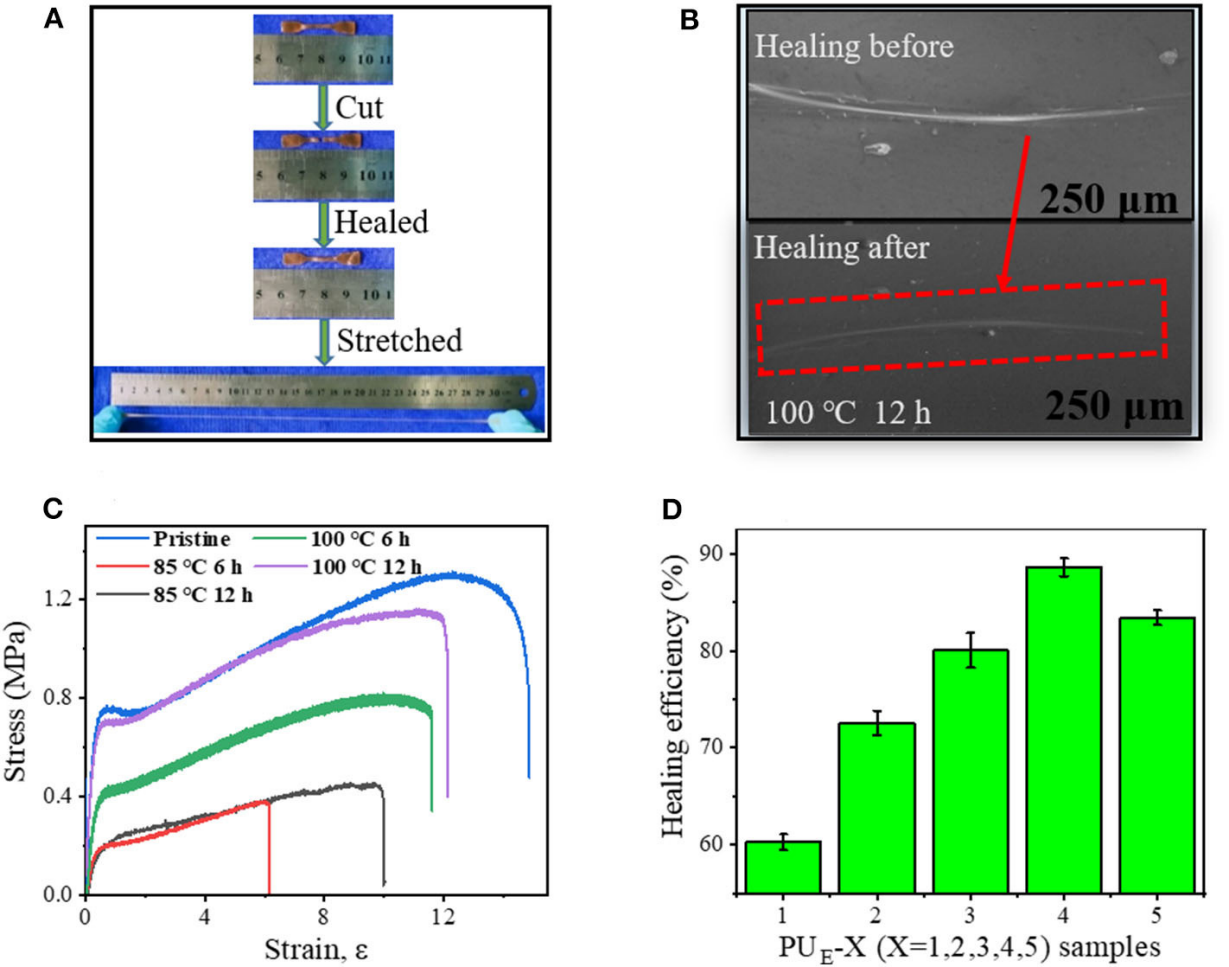
Self-healing mechanism and properties of PU_E_ samples. **(A)** Pictures of before and after healing, and the healed sample under stretching. **(B)** SEM images of the damaged surfaces before and after healing at 100°C for 12 h. **(C)** Strain—stress curves of the healed at 85 and 100°C for various time. **(D)** Healing efficiency of PU_E__S_amples with various cross-linker bcc content (healed at 100°C for 12 h).

To demonstrate the self-healing behavior, tensile tests before and after self-healing are performed. As shown in Figure 6A, the healed sample can sustain a large strain after healing at 100°C for 12 h, indicating the excellent self-healing ability of the PU_E_ samples. Figure 6C exhibits the typical stress–strain curves of PU_E_-4 healed at 85 and 100°C for various times. The self-healing efficiency is quantified by the ratio of the fracture stress of the healed to the pristine sample in Supplementary Equation 3. After being healed for at 85°C for 12 h, the healing efficiency is 34.9%. Significantly, the healing efficiency further increase to 62.8% and 88.4% after being healed at 100°C for 6 and 12 h, respectively. The self-healing fully demonstrates the reconstructing ability of network topologies and hydrogen bonds. Figure 6D shows the healing efficiencies of different PU_E_ samples. It is worth noting that the healing efficiency does not change monotonously with cross-linking density, and PU_E_-4 sample exhibits the highest efficiency. In general, higher crosslinking density indicates restricted chain mobility, which is unfavorable for wetting and diffusion of rubber chains at interface and consequently leading to poor healing behavior (Cao et al., 2019). However, a higher cross-linking density means more carbonate bonds, which indicates that more covalent linkages can be reformed at interface via transcarbonation exchange reaction. The competition of these two effects leads to the highest healing efficiency of PU_E_-4. To investigate the healing from microscopic scale, we prepare a scratch on a sample film and characterize the evolution of the scratch with scanning electron microscope (SEM). Figure 6B exhibits that the scratch on the film almost completely disappears after healing at 100°C for 12 h. This once again indicates the good self-healing behavior of the obtained PU_E_ samples.

## Conclusions

In summary, we have designed and fabricated a novel acid-degradable and self-healable vitrimer based on non-isocyanate PU_E_. Interestingly, the elastomer manifests a unique strain-rate–responsive yield phenomenon during stretching. The yield stress increases with increasing strain rate, as the increasing high strain rate (>0.004 s^−1^) tends to enhance the strain localization and promote yielding. Elevating the temperature can alter the networks topologies of the elastomer, which results in excellent reprocessing and self-healing behaviors. Simultaneously, the elastomer containing the dynamic networks can be hydrolyzed and decarbonated in the strong acid solution (HCl) to recovery 75 % of the pure di(trimethylolpropane) monomer. Therefore, we can envision that this non-isocyanate-based PU_E_ will manifest longer service life and decreased pollution to the environment.

## Data Availability Statement

All datasets generated for this study are included in the article/Supplementary Material.

## Author Contributions

HWu carried out experiment and wrote the manuscript. JW supervised the project and mainly revised the paper. All authors extensively reviewed the manuscript and approved the final version of the manuscript to be submitted.

## Conflict of Interest

The authors declare that the research was conducted in the absence of any commercial or financial relationships that could be construed as a potential conflict of interest. The reviewer LC declared a past co-authorship with one of the authors JW to the handling editor.
