# Supplementary material for: A Degradable and Self-Healable Vitrimer Based on Non-isocyanate Polyurethane
Source: Front Chem. 2020 Oct 16;8:585569. doi: 10.3389/fchem.2020.585569 (PMC7604760; doi:10.3389/fchem.2020.585569)
Supplement: Supplementary file 1 [file Table_1.DOCX]

Supplementary Material

**Cross-linking density:** Cross-linking density is calculated from the following formula:

ν=$\frac{E'}{3RT}$ (1)

where *T* is absolute temperature at *T_g_* + 30 ℃, *R* is the gas constant and *E'* is storage modulus.

**Tensile strength recovery efficiency:** Tensile strength recovery efficiency was quantified by ratio maximum fracture stress reprocessing before to after, as below:

Tensile strength recovery efficiency =$\frac{\sigma\left( reprocessing after \right)}{\sigma\left( reprocessing before \right)}$ (2)

**Self-healing efficiency:** The self-healing efficiency was quantified by ratio the fracture stress of the healed to the pristine sample, as below:

Healing efficiency =$\frac{\sigma\left( healed \right)}{\sigma\left( pristine \right)}$ (3)

**Table S1.** The feed ratios of PU_E_-X(X=1,2,3,4,5) samples.

| sample | mol % Ti^4+^ | bcc | Pre- PU_E_ |
| --- | --- | --- | --- |
| PU_E_-1 | 2 | 0.260 g, 0.860 mmol | 6.00 g, 0.860 mmol |
| PU_E_-2 | 2 | 0.312 g, 1.032 mmol | 6.00 g, 0.860 mmol |
| PU_E_-3 | 2 | 0.416 g, 1.376 mmol | 6.00 g, 0.860 mmol |
| PU_E_-4 | 2 | 0.467 g, 1.548 mmol | 6.00 g, 0.860 mmol |
| PU_E_-5 | 2 | 0.519 g, 1.720 mmol | 6.00 g, 0.860 mmol |

**Table S2.** Cross-linking density of PU_E_ samples.

| Samples | (*T_g_*+30℃)  *E'*(storage modulus) | *T* (*T_g_*+30℃) | Cross-linking density |
| --- | --- | --- | --- |
| PU_E_-1 | 1.6 | 266.15 | 0.000241 |
| PU_E_-2 | 2 | 273.15 | 0.000294 |
| PU_E_-3 | 2.69 | 277.15 | 0.000389 |
| PU_E_-4 | 3.92 | 280.15 | 0.000561 |
| PU_E_-5 | 12.94 | 285.15 | 0.001819 |

**Table S3.** Thermal characteristics of PU_E_ series samples determined by DMA.

| Samples *T_g_* (°C) | Storage modulus (MPa)  65 °C 80 °C | | Loss factor  65 °C 80 °C | |
| --- | --- | --- | --- | --- |
|  |  |  |  |  |
| PU_E_-1 -18  PU_E_-2 -23  PU_E_-3 -26  PU_E_-4 -30  PU_E_-5 -37 | 0.36 | 0.26 | 0.52 | 0.55 |
|  | 0.64 | 0.49 | 0.47 | 0.54 |
|  | 0.84 | 0.67 | 0.43 | 0.47 |
|  | 1.42 | 1.09 | 0.31 | 0.35 |
|  | 6.89 | 5.39 | 0.17 | 0.16 |

**Table S4.** Summary of mechanical properties of PU_E_-X(X=1,2,3,4,5) samples (measured at a stretching rate of 0.083 s^−1^).

| Samples | Tensile strength (MPa) | Strain at break (%) | Young’s modulus (MPa) | Toughness (kJ m^–3^) |
| --- | --- | --- | --- | --- |
| PU_E_-1 | 0.79±0.11 | 1909.37±51 | 0.011±0.02 | 12.20±0.04 |
| PU_E_-2 | 1.01±0.28 | 1818.06±17 | 0.009±0.03 | 14.32±2.42 |
| PU_E_-3 | 1.12±0.64 | 1734.95±136 | 0.012±0.03 | 13.45±5.21 |
| PU_E_-4 | 1.32±1.07 | 1481.53±57 | 0.021±0.07 | 15.47±4.75 |
| PU_E_-5 | 2.09±1.43 | 1342.51±121 | 0.029±0.09 | 20.57±7.89 |

**
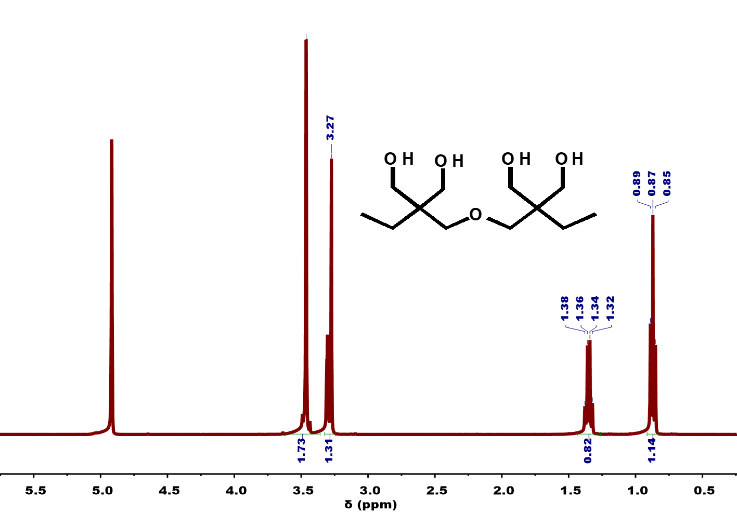
**

**Figure S1.** ^1^H NMR (CD3OD, AV III HD 400 MHz, Bruker) of di(trimethylolpropane) monomer.

**
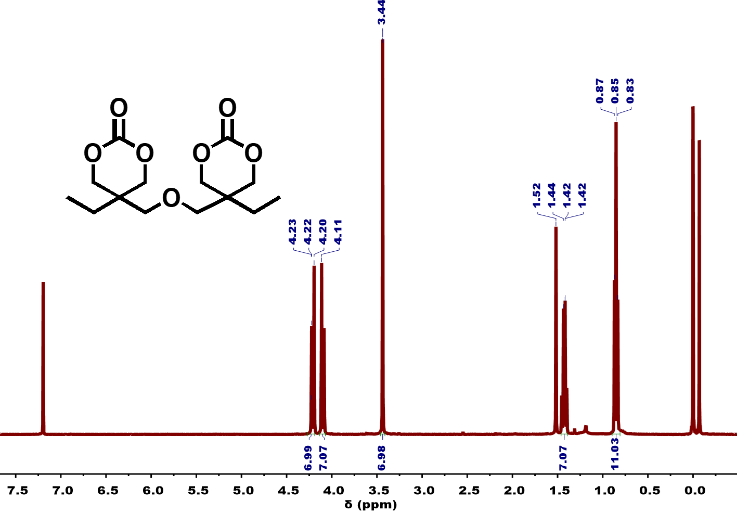
**

**Figure S2.** ^1^H NMR (CDCl3, AV III HD 400 MHz, Bruker) of bis(cyclic carbonate) (bcc).

**

**

**

**


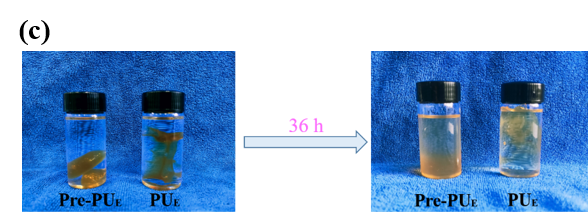


**Figure S3.** FT-IR characterization of **(a)** the prepared Pre-PU_E_ and **(b)** the cross-linked PU_E_ series samples. **(c)** Swelling tests of Pre-PU_E_ and PU_E_ for 36 h at room temperature.





**Figure S4.** Temperature dependent FT-IR spectra of PU_E_ upon heating from 10 ℃ to 150 ℃.

**

**











**Figure S5.** **(a)** The effect of cross-linker content on the dual-networks elastomer glass-transition temperature (*T_g_*). **(b)** The TGA curves of PU_E_ samples under N_2_ in the temperature range of room temperature to 600 °C at a temperature ramp rate of 10 °C min^-1^. **(c)** The temperature of maximum rate of weight loss *T_max_* and the percentage weight loss at *T_max_* are determined from the differential thermos-gravimetric (DTG) traces. **(d)** Dependence of the PU_E_ series samples loss factor (tan*δ*) on temperature.

**

**

**Figure S6.** Stress relaxation curves of PU_E_ samples. The samples are stretched to a strain of 50 % at 100 °C and then maintained for 12 min.





**Figure S7.** Dependence of the fracture strain on the various strain rates.


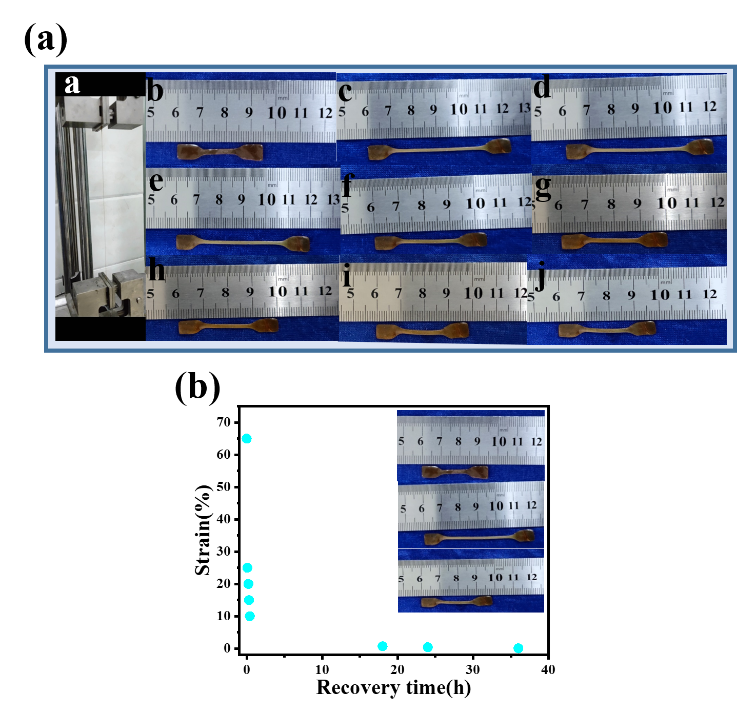


**Figure S8.** (a) Ultra-stretchable PU_E_ shape recovery after they are subjected to large deformations. (b) PU_E_ sample is elastic and deformation strain gradually recovery as a function of time.


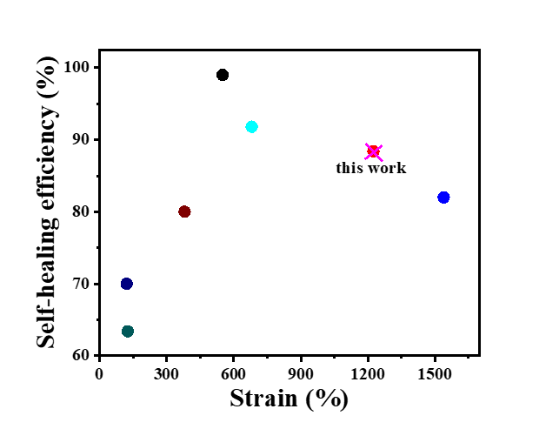


**Figure S9.** The performance comparison between this material and the existing literatures PU elastomer


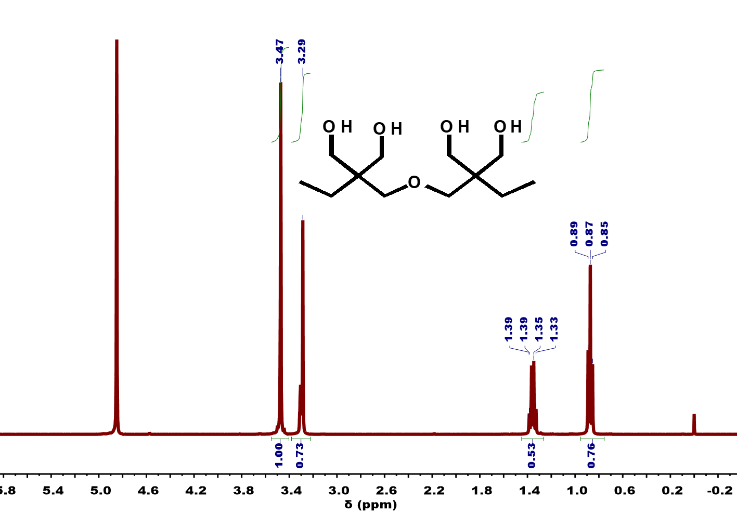


**Figure S10.** ^1^H NMR (CD3OD, AV III HD 400 MHz, Bruker) of the acid degradation recovery di(trimethylolpropane) monomer.





**Figure S11.** The FT-IR spectroscopy of reprocessing PU_E_ samples.





# Figure S12. The ratio of maximum fracture stress reprocessing before to after.
